# Plant Growth Promotion Diversity in Switchgrass-Colonizing, Diazotrophic Endophytes

**DOI:** 10.3389/fmicb.2021.730440

**Published:** 2021-11-12

**Authors:** Sara Gushgari-Doyle, Marcus Schicklberger, Yifan V. Li, Robert Walker, Romy Chakraborty

**Affiliations:** Climate and Ecosystem Sciences, Earth and Environmental Sciences Area, Lawrence Berkeley National Laboratory, Berkeley, CA, United States

**Keywords:** endophyte, nitrogen-fixation, switchgrass, plant growth promoting (PGP) bacteria, diazotroph

## Abstract

Endophytic nitrogen-fixing (diazotrophic) bacteria are essential members of the microbiome of switchgrass (*Panicum virgatum*), considered to be an important commodity crop in bioenergy production. While endophytic diazotrophs are known to provide fixed atmospheric nitrogen to their host plant, there are many other plant growth-promoting (PGP) capabilities of these organisms to be demonstrated. The diversity of PGP traits across different taxa of switchgrass-colonizing endophytes is understudied, yet critical for understanding endophytic function and improving cultivation methods of important commodity crops. Here, we present the isolation and characterization of three diazotrophic endophytes: *Azospirillum agricola* R1C, *Klebsiella variicola* F10Cl, and *Raoultella terrigena* R1Gly. Strains R1C and F10Cl were isolated from switchgrass and strain R1Gly, while isolated from tobacco, is demonstrated herein to colonize switchgrass. Each strain exhibited highly diverse genomic and phenotypic PGP capabilities. Strain F10Cl and R1Gly demonstrated the highest functional similarity, suggesting that, while endophyte community structure may vary widely based on host species, differences in functional diversity are not a clearly delineated. The results of this study advance our understanding of diazotrophic endophyte diversity, which will allow us to design robust strategies to improve cultivation methods of many economically important commodity crops.

## Introduction

Nitrogen (N) is an essential component of biomolecules such as proteins and nucleic acids, and is consequently a key element for life and cell development. Availability of N, along with phosphorus, is often the limiting factor for plants, thereby significantly reducing plant growth and biomass yield (Muir et al., 2001; Zhao et al., 2005). N is available to plants either as N fixed from the atmosphere by N_2_-fixing, plant-associated microorganisms or from synthetic inputs such as mineral fertilizer. To alleviate N limitation in agricultural practice, copious amounts of mineral N fertilizer are often added to maximize plant yields. While this practice has been partly responsible for the “green revolution,” it has come at high environmental and economic costs. The fertilizer industry utilizes 1.2% of the world’s energy resources and more than 90% of this is used for mineral N fertilizer production (U. S. Energy Information Administration, 2010), which also represents about 5% of global natural gas consumption. Additionally, fertilizer amendment to overcome N limitation destabilizes native soil ecosystems, produces greenhouse gases, and introduces carcinogens to the environment (Ayanaba et al., 1973; Chen et al., 2008; Snyder et al., 2009; Li et al., 2014), and N-based fertilizer runoff and leaching of nitrate from cropland create water quality problems such as eutrophication (Carpenter et al., 1998). Due to the unavoidable reliance of our society on plants for food and biofuel production, it is imperative to harness ecologically friendly practices to develop more productive, resilient, and sustainable crops.

In natural ecosystems, plants have developed strong relationships with microorganisms to cope with low availability of essential nutrients like N and other environmental stressors (Steenhoudt and Vanderleyden, 2000; Franche et al., 2009; Kumar and Verma, 2018; Grady et al., 2019). Microbially mediated nitrogen fixation, the reduction of atmospheric N_2_ to ammonia by diazotrophic bacteria, is the principal natural mechanism by which N enters terrestrial ecosystems. While nodule-forming legumes are the most historically known for the N_2_-fixation symbiosis (Franche et al., 2009; Masson-Boivin et al., 2009), there are many instances of N_2_-fixing (diazotrophic) bacteria that are able to fix atmospheric N_2_ without forming this relationship with a host plant (Santi et al., 2013). Endophytic bacteria with N_2_-fixing function that colonize roots, stems, and leaves of plants have been identified in several plants unable to form symbiotic organs (Hurek et al., 2002; Lowman et al., 2015). Microbial community diversity, and therefore competition for substrates and nutrients, is comparatively lower in the endosphere than in rhizosphere communities (Jin et al., 2014). Additionally, endophytes are generally more protected from environmental stress once colonized in the plant tissue (Hallmann et al., 1997) and may be left undeterred inside plant tissues to develop a close, mutualistic relationship with the host plant. Therefore, optimizing interactions between plants and N_2_-fixing endophytes provides a more sustainable method of providing adequate amounts of assimilable N to host crops (Olivares et al., 2013). The resulting shift away from synthetic fertilizer utilization would reduce the carbon footprint and improve sustainability of feedstock production while diminishing contamination of agricultural lands.

To optimize diazotrophic endophyte interactions, it is important to comprehensively understand the diversity of diazotrophic endophytic plant growth-promoting (PGP) traits, which is likely unique to each plant host genotype (Reinhold-Hurek and Hurek, 2011). Switchgrass, a high-producing, perennial C4 grass known for its low nutrient requirements (Lewandowski et al., 2003), is the ideal candidate for investigating diazotrophic endophytes due to its widespread utilization in North America as a bioenergy feedstock and energy markets that are dependent on a low greenhouse gas footprint (Lewandowski et al., 2003; Lowman et al., 2015). In addition to low nutrient requirement, switchgrass exhibits robust resistance to environmental perturbations, high biomass yield, and high tolerance to stressors (e.g., drought, temperature, and metals) (Lewandowski et al., 2003). Tobacco (*Nicotiana tabacum*) is also a bioenergy feedstock candidate due to the substantial land-use dedicated to tobacco cultivation coupled with increasing subsidy restrictions on the crop (Grisan et al., 2016). Growth and environmental resilience of both crops are bolstered by their microbiomes, including the rhizosphere and endophyte communities (Brejda et al., 1998; Afzal et al., 2017; Begum et al., 2018; Jiao et al., 2020). While the taxonomic diversity of switchgrass endophytes has been studied (Kim et al., 2012; Xia et al., 2013; Bahulikar et al., 2014; Wang et al., 2015; Singer et al., 2019), the functional diversity of PGP traits among diverse species of switchgrass-colonizing endophytes is unclear.

To address this knowledge gap, we present the isolation of diazotrophic endophytes able to colonize switchgrass plant tissues and phenotypic characterization and thorough genomic comparison of three novel switchgrass-colonizing endophytes in the genera *Raoultella*, *Azospirillum*, and *Klebsiella*. The *Raoultella* and *Azopirillum* strains are the first of their genera to be reported as isolates in the switchgrass endosphere, while *Klebsiella* was only recently reported as a switchgrass endosphere isolate (Jones et al., 2021). The genotype to phenotype analysis herein elucidates the diversity of PGP genes and functions present in N_2_-fixing, switchgrass-colonizing endophytes. The results of this study will improve understanding of the characteristics and diversity of the diazotrophic endophytic community found in switchgrass to more comprehensively understand, and ultimately manipulate, the N_2_-fixing microbiome of this economically important grass species.

## Materials and Methods

### Isolation of Switchgrass-Colonizing Endophytes

Switchgrass (*Panicum virgatum* EG1101 cultivar) and tobacco (*Nicotiana tabacum*) were grown from seeds purchased from Outsidepride.com Inc. (Independence, OR, United States) for 6 weeks in a laboratory greenhouse (Berkeley, CA, United States) in biological triplicate. One 6-week-old plant of each variety was harvested and surface sterilized [sterilized water, 75% (v/v) ethanol wash, sterilized water wash (4×), 60% (v/v) bleach wash, sterilized water wash (6×)]. Leaf and root tissues from the sterilized plants were used to isolate endophytic microbes. Prior to mortar and pestle maceration, surface-sterilized and unsterilized leaves and roots were placed on different rich media to determine the effectiveness of the sterilization procedure. An abundance of fungal and bacterial epiphytes was observed on all growth media with unsterilized roots and leaves from both switchgrass and tobacco plants, but no growth was observed on media with surface-sterilized roots and leaves. The surface-sterilized roots and leaves were then macerated and used as inoculum for 48-well plates containing modified Jensen’s media (Supplementary Table 1) or HGB media (Jensen, 1942; Holguin et al., 1992) with a variety of carbon substrates (10 mmol l^–1^) such as sucrose, fructose, malate, succinate, and acetate. A serial dilution of the inoculum was carried out along the *X* axis of the 48-well plates in duplicate, with one set containing 2% agar. Distinct colonies were developed after 10–14 days incubation in the dark at room temperature. Colonies from the highest dilutions were picked, restreaked on plates containing the same growth medium and 2% agar, then transferred into liquid media.

### Verification of Nitrogen Fixation

#### Growth on N-Free Media

Endophytic isolates were grown to mid-log phase and centrifuged at 4,000 *g* for 3 min to concentrate cells. The pellets were washed and resuspended in 30 mmol l^–1^ phosphate buffer. 1 mL of this inoculum was transferred to 10 mL anaerobic culture tubes (crimp-cap sealed with butyl rubber stoppers) containing N-free NFb media (Döbereiner and Day, 1976) to evaluate capacity of the isolates for N_2_-fixation. After 1 week, isolates grew and formed a pellicle in the tubes. The tubes containing the isolates were tested for active N_2_-fixation using the acetylene reduction assay (Supplementary Figure 1).

#### Acetylene Reduction Assay

Isolates demonstrating active N_2_-fixation were screened with the acetylene reduction assay (Dilworth, 1966) to confirm diazotrophy. 48 h after inoculation, 5 mL >99.2% acetylene gas (Praxair, United States) was amended to the cultures. Ethene production was measured via gas chromatograph equipped with a thermal conductivity detector (model GC-8A, Shimadzu, Japan) and a 80/100 Hayesep T column (2.50 m × 1.8 in, Supelco, United States) with detector temperature 120°C, column temperature 70°C, currency 140 mA, and sampling rate of 1 per 100 milliseconds. Three isolates exhibited high levels of N_2_-fixation and were selected for further analysis.

#### *Nif* Primers

Several set of *Nif* primers were evaluated for *nifH* gene amplification based on previous literature including PolFR, F2, Kadino, nifH3, and R6 (Gaby and Buckley, 2012). The PolFR primers (PolF – 5′ TGCGAYCCSAARGCBGACTC 3′, PolR – 5′ ATSGCCATCATYTCRCCGGA 3′) were selected for *nifH* amplification (Poly et al., 2001).

### Species Identification and Whole Genome Sequencing

Genomic DNA from bacterial isolates was extracted using a PureLink Genomic DNA Mini Kit (Invitrogen, United States) following the manufacturer’s protocol. 16S rRNA genes were amplified using the eubacterial primers 27F (AGA GTT TGA TCC TGG CTC AG) and 1492R (ACG GCT ACC TTG TTA CGA CTT) (Integrated DNA Technologies, Inc., United States). Sanger sequencing of 16S rRNA PCR product was performed at University of California Berkeley DNA Sequencing Facility. The PCR products were sequenced using the internal primers 27F and 1492R. Consensus sequences (1200–1400 base pairs) from forward and reverse sequences were generated using Geneious (version 9.1.3) and deposited in Genbank under accession numbers MZ747095-MZ747097. The NCBI and SILVA databases were used for bacterial isolate classification (Sayers et al., 2011; Quast et al., 2013).

Whole genome sequencing was performed on the PacBio RS sequencing platform at the Joint Genome Institute (JGI, Berkeley, United States). Raw reads were assembled using HGAP (version: 2.2.0.p1) (Chin et al., 2013). Coding sequence prediction, gene identification, and annotation were performed as previously described (Schicklberger et al., 2015). Genome sequences for isolates R1C, F10Cl, and R1Gly were deposited in GenBank^1^ under the BioProject accession numbers PRJNA257885, PRJNA257883, and PRJNA254925, respectively. The genome of isolate R1Gly has been announced previously (Schicklberger et al., 2015).

### Comparative Genome Analysis

Genome comparison of isolates F10C1, R1C, and R1Gly was performed using various functions in IMG/MER (Chen et al., 2019), including KEGG pathway search (KEGG release 95.0), Phenotype, and function search. COG (clusters of orthologous groups) comparison was performed in IMG/MER using the Compare Functions tool.

Putative plasmids were identified by selecting scaffolds smaller than 500 kbp with differing GC content for further investigation. Putative plasmids were compared against the plasmid database PLSDB (v.2020_03_04) using the Mash pair-wise distance function (Ondov et al., 2016; Galata et al., 2019) and either confirmed with 100% identity to a known plasmid, identified as a related plasmid (pair-wise distance < 0.1) (Galata et al., 2019; Jesus et al., 2019), identified as a putative plasmid (*p*-value < 1e-30, 0.1 < pair-wise distance < 0.25), or discarded as a putative plasmid.

### Characterization of N_2_-Fixing Isolates

Several laboratory characterization assays were performed based on the results of genome comparison and knowledge of common PGP characteristics to verify the results of *in silico* genome comparison.

#### Carbon Utilization Assay

To determine the organic carbon compounds utilizable as electron donor and carbon source by each N_2_-fixing isolate, a growth assay was performed. Briefly, cells were washed and normalized to an OD_600_ of 0.5 and used as inoculum (10% v/v). The assay was performed in 96-well plates with basal growth medium with 10 mmol l^–1^ of carbon source with a total volume of 0.2 ml per well. Plates were incubated in the dark at 30°C with shaking for 48 h. Carbon sources evaluated include sucrose, fructose, glucose, ribose, xylose, lyxose, cellobiose, arabinose, mannose, rhamnose, maltose, malic acid, citric acid, lactic acid, benzoic acid, acetic acid, oxalic acid, succinic acid, fumaric acid, galacturonic acid, glycolic acid, mucic acid, phytic acid, nicotinic acid, and urea.

#### Indole-3-Acetic Acid Assay

To experimentally validate indole-3-acetic acid (IAA) biosynthesis, a colorimetric assay and HPLC quantification were used. Isolates were grown in RCH2 liquid medium containing 5 g l^–1^ glucose, 25 mg l^–1^ yeast extract, 200 mg l^–1^ L-tryptophan at pH 7.2. Briefly, cells were normalized to an OD_600_ of 0.5 for use as inoculum (10% v/v) and incubated in the dark at 30°C for 43 h.

Samples for the colorimetric assay using the Salkowski Method were taken at hours 27 and 43 and analyzed as previously described (Bric et al., 1991). Samples for high-performance liquid chromatography (HPLC) were taken at 4 timepoints: 0, 20, 27, and 43 h. IAA was measured via HPLC (LC 1260 Series, Agilent Technologies) equipped with a ZORBAX Eclipse Plus C18 column (4.6 × 100 mm, Agilent Technologies) and UV/VIS detector (Polygen, Denmark) at 220 nm in a methanol/water (80:20 vol:vol) mobile phase as previously described (Hariharan et al., 2014).

#### Chitinase Activity Assay

The isolates were also evaluated for chitinolytic activity on agar plates as previously described (O’Brien and Colwell, 1987; Sampson and Gooday, 1998). Briefly, isolates were grown to mid-log phase in liquid R2A medium and washed and resuspended in PBS buffer. Resuspended isolates were spotted on solid chitin agar and incubated for 72 h at 30°C. Positive chitinase activity was identified with a change of color (from white to blue) around the isolate colony.

#### Cellulase Activity Assay

The isolates were evaluated for cellulase enzyme activity in liquid medium as previously described (Nyyssönen et al., 2013). Briefly, log-growth stage cells were washed and inoculated in liquid R2A + 0.1% (w/v) AZCL-HE-Cellulose (Megazyme, Ireland) in the dark at 30°C for 5 days in experimental triplicate. OD590 was measured at day five to allow for natural release of cellulase enzymes via export or cell lysis. *Cellulomonas pakistanensis* XG116, obtained from our internal culture collection, was used as the positive control. Uninoculated medium and *Pseudomonas fluorescens* strain N2E3, obtained from our internal culture collection, were used as negative controls.

#### Hydrogen Cyanide Assay

The isolates were evaluated for hydrogen cyanide (HCN) biosynthesis in liquid medium as previously described (Zlosnik and Williams, 2004; Rijavec and Lapanje, 2016). Briefly, log-growth stage cells were washed and inoculated (10% v/v) in liquid glycine-supplemented media and incubated in the dark at 30°C for 24 and 48 h with shaking at 120 rpm. Aliquots (100 μl) were centrifuged at 13,000 rpm for 2 min and 50 μl supernatant was transferred into a 96-well microtiter plate with 140 μl Milli-Q water and 10 μl methemoglobin reagent. Plates were incubated in the dark for 30 min and absorbance was measured at 424 nm. OD of cultures at 24 and 48 h was also measured at 600 nm. *Paraburkholderia fungorum* strain MS9-19, obtained from our internal culture collection, was used as a positive control. Uninoculated blank medium was used as a negative control. All conditions were performed in experimental triplicate.

#### Siderophore Production Assay

Siderophore production was qualitatively evaluated using a colorimetric, overlay-chrome azurol S (CAS) assay as previously described (Schwyn and Neilands, 1987; Pérez-Miranda et al., 2007). Inoculated 96-well plates were incubated at 30°C for 72 h, overlaid with CAS, and incubated overnight before colorimetric analysis. A resulting purple color indicated catechol-type siderophore production, yellow indicated hydroxamate-type siderophore production, red-orange indicated a mix of different siderophore types, and blue indicated the absence of detected siderophore production.

#### Infection of Switchgrass With Strain R1Gly

Strain R1Gly, which was isolated from tobacco, was inoculated into sterilized switchgrass seeds to determine if the isolate could establish endophytic colonization in switchgrass as evaluated by *gfp* fluorescence under *nifH* reporter control. To introduce fluorescence, the *gfp* reporter gene was introduced downstream of *nifH* under *nifH* reporter control. Briefly, primers bac_nifH_for and nifD_rev were used to amplify a nifHD fragment from the genome of strain R1Gly via primer walking (Minerdi et al., 2001) and Sanger sequenced to obtain DNA sequences needed for homologous recombination. The *gfp* gene was amplified from pMQ97 (Shanks et al., 2006) using *gfp*-flanking primer set gfp_for and gfp_rev. The gfp gene and upstream and downstream fragments for recombination were fused using primers pMQ_nifHR1Gly_up_for and pMQ_nifHR1Gly_down_rev, then ligated into the linearized vector pMQ150 to generate pMQnifH:gfp. The pMQnifH:gfp shuttle vector was transformed into *E. coli* WM3064 and selected for on LB plates containing 50 μg ml^–1^ kanamycin and 50 μg ml^–1^ diaminopimelic acid to be used as the donor strain. Selected strains were confirmed to contain the fragment of interest via amplification using primers pMQ_test_for and pMQ_test_rev. After positive confirmation, shuttle vector pMQnifH:gfp was then transferred into strain R1Gly via conjugation in 1/5 LB growth medium at 37°C. A 150 μl aliquot of undiluted, 1:5 (v/v), and 1:25 (v/v) culture was streaked on LB plates containing 50 μg ml^–1^ kanamycin and incubated for 2 days at 37°C. Resulting R1Gly-nifH:gfp colonies were picked and a second homologous recombination was performed to remove the pMQ vector background and grown in LB growth medium. Then, 150 μl aliquots of culture were plated on RCH2 plates containing 10% (m/v) sucrose for counterselection. Colonies were picked and nifH:gfp mutants confirmed with primers bac_nifH_for and nifD_rev. Confirmed mutants were grown in LB and in nitrogen-deficient medium with N_2_/CO_2_ (80:20 v/v) headspace and visualized via fluorescence microscopy with a blue filter for *gfp* signal detection. Nitrogen fixation activity in nitrogen-deficient medium cultures was confirmed via the acetylene assay. Confirmed mutants were preserved in glycerol stocks for further use. A list of primers used herein is available in Supplementary Table 2.

Pre-sterilized switchgrass seeds were germinated under aseptic conditions on agar plates for 7 days and subsequently infected with 1 μl of active cultures of the fluorescent tagged strains. The plant hosts were grown in N_2_ deficient medium with 2% agar for 2 months. Cross sections for fluorescence pictures were prepared by embedding the plant tissue in a Styrofoam cube and cutting thin sections with a utility blade. The pictures were taken via fluorescence microscopy (Axioskop 2, Zeiss, United States) with a MicroFire camera (Optronics, United States). At the end of the experiment, plant host leaves, and roots wet weights were measured to quantify plant growth. The experiment was performed with three replicates per treatment. The Student’s *t*-test was used to determine is wet weights were significantly different between R1Gly-infected switchgrass and the uninfected negative control.

## Results

### Isolation and Identification of Diazotrophic Endophytes

Three hundred (300) isolates were obtained from surface sterilized and macerated roots and leaves of switchgrass and tobacco plants and evaluated for N_2_-fixing capacity. Of these, about two dozen exhibited diazotrophy, and three isolates that exhibited high levels of N_2_-fixation designated strains *Klebsiella variicola* strain F10Cl and *Azospirillum agricola* strain R1C (isolated from switchgrass), and *Raoultella terrigena* strain R1Gly (isolated from tobacco) were selected for further characterization.

The 16S rRNA gene was sequenced for the three selected isolates. Genetically closest relatives were identified using the NCBI and SILVA databases (Table 1).

**TABLE 1:** 16S rRNA gene MegaBLAST results for three diazotrophic endophyte isolates.

Isolate	Closest species match	NCBI identity (%)	Silva identity (%)	Accession	Reference
F10Cl	*Klebsiella variicola* strain GJ3	99.9	99.9	SAMN05361855	Di et al., 2017
R1C	*Azospirillum agricola* strain CC-HIH038	98.0	97.9	NR148768	Lin et al., 2016
R1Gly	*Raoultella terrigena* strain ATCC 33257	99.6	99.4	NR114503	Drancourt et al., 2001

### Infection of Switchgrass With Strain R1Gly

**FIGURE 1:**
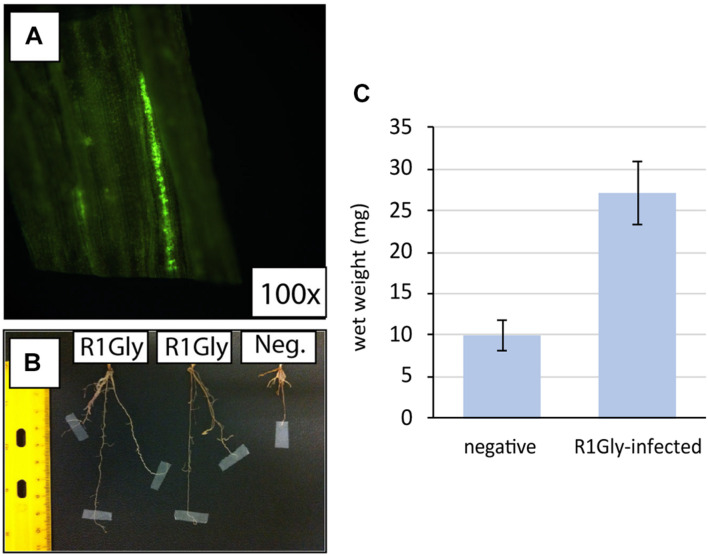
**(A)** Fluorescence of *nifH::gfp* strain R1Gly in the leaf section of re-infected switchgrass (100×). **(B)** Root lengths and **(C)** below ground surface wet masses of R1Gly-infected switchgrass (R1Gly) versus sterile switchgrass (negative). Error bars represent one standard deviation of biological triplicates.

To verify strain R1Gly could also establish an endophytic relationship with switchgrass, R1Gly was infected into sterilized switchgrass seeds with a fluorescence-labeled *nifH:gfp* R1Gly mutant (Figure 1 and Supplementary Figure 1). The cells were observed to fluoresce (indicating active *nifH* expression) in the leaf section of reinfected switchgrass, as observed in the R1Gly *nifH:gfp*-infected grass leaf (Figure 1A). When compared to uninfected, sterilized switchgrass, the R1Gly *nifH:gfp*-infected switchgrass grew significantly larger root structures (*p* = 3.31 × 10^–6^) after 2 months of incubation (Figures 1B,C).

### Genome Architecture of Diazotrophic Endophytes

Whole genome sequencing was performed on F10Cl, R1C, and R1Gly. The permanent draft genomes were used for identification of genes associated with PGP characteristics. Genome sizes ranged from 5.7 Mb (F10Cl, R1Gly) to 7.7 Mb (R1C) with varying GC content and protein coding genes (Supplementary Table 3). COG (cluster of orthologous groups) comparisons among the three strains revealed a core set of 1,369 COGs common to all three isolates. R1C, F10Cl, and R1Gly also possessed unique COGs (527, 70, and 58, respectively) that were not present in the other strains. The greatest orthology was observed between strains F10Cl and R1Gly (628 COGs) - both strains belonging to *Alphaproteobacteria* but isolated from difference sources. The lowest COG synteny was between R1C and R1Gly (49 COGs).

Plasmid pNDM-MAR was found and confirmed in strain F10Cl (Villa et al., 2012), and three and two putative plasmids with pair-wise distances from known plasmids < 0.25 were found in strains R1Gly and R1C, respectively (Supplementary Table 4).

**FIGURE 2:**
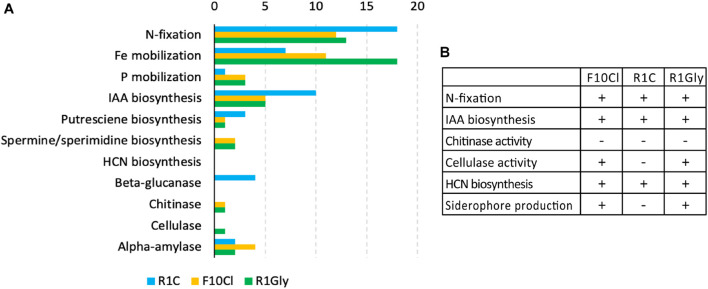
**(A)** Numbers of genes associated with select PGP traits found in each isolate genome: R1C (blue), F10Cl (orange), and R1Gly (green). **(B)** Result summary for laboratory characterization assays. (+) indicates a positive result (activity observed) and (–) indicates a negative result (not observed).

The three isolate genomes were evaluated for genes associated with PGP activities (Figure 2A). In addition to comparison of N_2_-fixation nif-operon (including *nifH*) (Supplementary Figure 2) strain R1Gly had a second nitrogenase gene *anfH* and R1C had additional N_2_-fixation genes *fixGHI*. The genome of strain R1Gly possessed the most Fe mobilization genes (18) while strain R1C possessed the least (7). Strain R1C possessed the most genes for IAA biosynthesis. Strains F10Cl and R1Gly both possessed genes for chitinase and cellulase, while R1C possessed several β-glucanase genes. Gene lists of PGP traits for the three isolates can be found in the Supplementary Table 5. In addition to genes encoding PGP traits, strain R1C possessed genes for flagellar biosynthesis while the other two strains did not.

### Linking Genotype to Phenotype via Laboratory Characterization of Isolates

**FIGURE 3:**
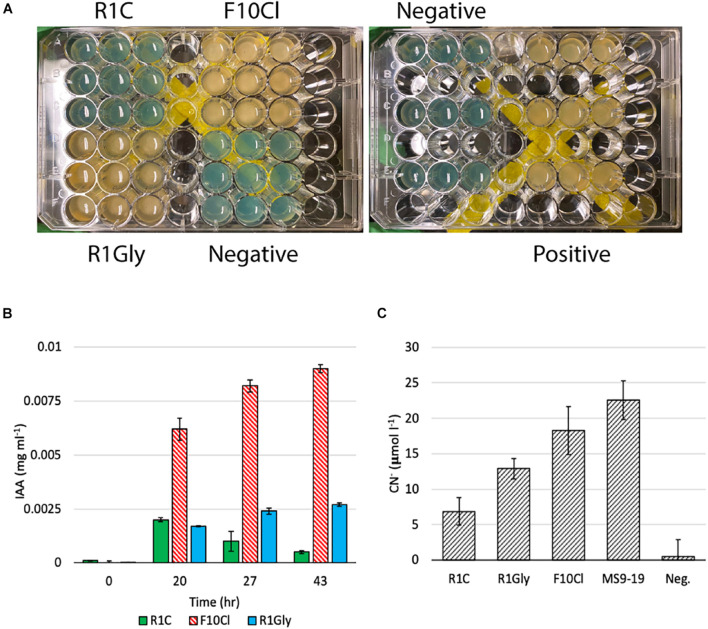
**(A)** Results of the qualitative siderophore O-CAS assay. Yellow color indicates production of hydroxamate-type siderophore and blue color indicates no siderophore production detected. *Pseudomonas marginalis* strain MS5-19 was utilized as a positive control. **(B)** Indole-3-acetic acid (IAA) production by strains R1C, F10Cl, and R1Gly over 43 h with tryptophan amended. **(C)** HCN biosynthesis measured as aqueous concentration of CN^–^ (μmol l^–1^). *Paraburkholderia fungorum* strain MS9-19 was utilized as a positive control. Error bars represent one standard deviation of three biological replicates.

Based on the results of the genomic analyses, several laboratory assays were performed to phenotypically verify predicted functions, the results of which are summarized in Figure 2B. The capacity of 25 plant-associated organic carbon compounds to support growth of each isolate were evaluated (Supplementary Table 6). Strain F10Cl demonstrated growth on the most substrates (21) and R1C the least (9). Additionally, siderophore and IAA biosynthesis, two functions commonly associated with PGP bacteria, were measured in all three isolates. Strains F10Cl and R1Gly possess genes for siderophore biosynthesis, which was confirmed experimentally with both strains producing hydroxamate-type siderophores (Figure 3A). IAA synthesis via a tryptophan-dependent pathway was experimentally confirmed in all three isolates (Figure 3B). HCN biosynthesis was observed in all three isolates, with strain F10Cl demonstrating the most HCN biosynthesis and R1C the least (Figure 3C). Chitinolytic activity was evaluated in all three isolates, but none of the isolates demonstrated this function (Supplementary Figure 3). Cellulolytic activity was observed in strains R1Gly and F10Cl (Supplementary Figure 4).

## Discussion

There are numerous endophyte functions that have been shown to promote plant growth and total primary productivity. Nutrients can be supplied to plants through biological N_2_-fixation or by the mobilization of nutrients, such as iron, zinc, and phosphorus (Rosenblueth and Martínez-Romero, 2006; Compant et al., 2010). This exchange can be vital in maintaining plant resilience, especially in conditions of environmental stress (Santoyo et al., 2016). Endophytes also contribute to plant health through the production of growth regulation hormones such as polyamines, cytokinins, auxins, and gibberellins.

### Nutrient Mobilization

As N_2_-fixation screening was performed in the selection of isolates for comparison in this study, all strains harbor a N_2_-fixation operon. The Fe-Mo nitrogenase subunit coding *nifH* gene is present and genomic context is highly orthologous among the strains (Supplementary Figure 2). Strain R1Gly has a second nitrogenase gene coding for the Fe-only nitrogenase AnfH. Additionally, in the genome of strain R1C, we encountered the N_2_-fixation operon FixGHI, an operon commonly found in N_2_-fixing symbionts (Kahn et al., 1989; Mandon et al., 1993). The genome of strain F10Cl only contained the Fe-Mo-type nitrogenase. Researchers have demonstrated that, in switchgrass, 16% of total plant N in the first 6 months of growth was obtained from bacterial N_2_-fixation, and hypothesized an increase in plant nitrogen from bacterial N_2_-fixation in mature plants (Keymer and Kent, 2014). The diversity in N_2_-fixation operons among the isolates suggests varying contribution of different endophytes to N_2_-fixation under varying conditions. Further studies are required to investigate this hypothesis.

Endophytic bacteria require iron for numerous cellular functions (e.g., N_2_-fixation) and can also mobilize iron for their plant hosts. Particularly, some bacteria produce siderophores which can chelate iron in iron-limited conditions and supply it to plants, promoting plant growth (Ahmed and Holmström, 2014). In the genomes of all three isolates, we encountered the siderophore-transporting complex TonB-ExbB-ExbD (Braun, 1995). Strains F10Cl and R1Gly possess genes for siderophore biosynthesis, which was confirmed experimentally with both strains producing hydroxamate-type siderophores, connecting genotype to phenotype (Figure 3A).

Solubilization of other trace nutrients including zinc, phosphorus, and potassium can also be a key benefit of plant growth promoting bacteria. Mechanisms of bacterial mineral and nutrient solubilization include production of chelating ligands, secretion of phytohormones and organic acids, and proton extrusion (Fasim et al., 2002; Saravanan et al., 2004). All strains demonstrated genomic capacity for at least one of these mechanisms.

### Plant Growth Hormone Regulation

Endophytes can contribute to plant growth both by producing plant growth hormones and repressing stress responses (Santoyo et al., 2016). For example, production of the auxin indole-3-acetic acid (IAA) by PGP bacteria has been shown to improve plant growth and crop yield (Malhotra and Srivastava, 2008; Sant’Anna et al., 2011). Auxins such as IAA are also likely to contribute to negative regulation of pathogen resistance, making the plant more susceptible to colonization (Shah, 2009; Spaepen and Vanderleyden, 2011). There are multiple known bacterial IAA biosynthetic pathways (Spaepen et al., 2007). All three isolate genomes contain some genes involved in these pathways, but only the genomes of strains F10Cl and R1Gly possess a full IAA biosynthetic pathway of tryptophan transformation via the indole-3-pyruvate pathway (Spaepen et al., 2007) according to genomic analysis via IMG/MER (with putative possession of the final catalytic enzyme based on NCBI BLAST). However, IAA synthesis via a tryptophan-dependent pathway was experimentally confirmed in all three isolates (Figure 3B), suggesting that there is unexplored diversity in IAA biosynthesis genes, missing information resulting from incomplete genomes, or an undiscovered tryptophan-dependent IAA biosynthesis pathway.

Bacterial production of the plant hormones spermine, spermidine, and putrescine have also been shown to promote plant growth (Perrig et al., 2007; Xie et al., 2014). All three strains possess ornithine decarboxylase for putrescine synthesis from ornithine, and strains F10Cl and R1Gly possess genes for spermine and spermidine biosynthesis. All isolates had numerous transporters for the plant growth hormones.

### Pathogen Resistance

Endophytes have been shown to trigger an immune response in plants that leads to a higher phytopathogen tolerance termed induced systemic resistance (ISR) (Zamioudis and Pieterse, 2012). Factors identified to be responsible for ISR include flagella, antibiotics, salicylic acid, siderophores, N-acyl-homoserine lactones, and lipopolysaccharides (van Loon et al., 2008; Shah, 2009; Bordiec et al., 2011). In addition to siderophore biosynthesis and transport, which have already been discussed, the isolates possess genes for phenazine, isopenicillin N, salicylic acid, and lipopolysaccharide biosynthesis. Strain R1C possesses flagellar genes while the other two isolates do not. HCN production has also been shown to suppress the growth of plant pathogens and mobilize phosphorus (Rijavec and Lapanje, 2016) and lytic enzymes such as β-glucanase, chitinase, and amylase are known to act as biological control agents, inhibiting fungal pathogen growth (Nagarajkumar et al., 2004). HCN biosynthesis activity was observed in all three isolates, but the hcnABC operon was not found in any genomes, suggesting unknown diversity in HCN biosynthesis genes. Several lytic enzymes were observed in each genome such as chitinase (F10Cl, R1Gly), β-glucanase (R1C), cellulase (F10Cl, R1Gly), and α-amylase (all). Cellulase activity was experimentally confirmed in both F10Cl and R1Gly, connecting genotype to phenotype. However, the laboratory assay for chitinase activity yielded negative results for all strains. It is possible that the genes were misannotated or there is an undiscovered chitinase nutrient dependency in these strains. The results presented herein suggest a diversity of pathogen-suppressing functions in switchgrass-colonizing endophytes as well as future research directions in improved annotation of PGP genes.

Diazotrophic endophytes can increase the resilience of feedstock crops, such as switchgrass, and have the capacity to provide assimilable N to host plants, diminishing the dependence of agriculture on synthetic mineral N fertilizer. In this study, we isolated diazotrophic endophytes from switchgrass and tobacco, demonstrated the PGP traits of these strains, and performed genomic comparison analyses to evaluate the capacities of three distinct bacterial strains. The tobacco-derived strain, R1Gly, was shown to colonize the switchgrass endosphere, demonstrating monocot colonization by a dicot-derived bacterial endophyte. While all strains are categorized as diazotrophic endophytes, each strain exhibits highly diverse PGP capabilities. Strain R1C possessed biocontrol-associated β-glucanase genes and additional N-fixation genes *fixGHI*, strain F10Cl exhibited the highest biosynthesis activity of both IAA and HCN as well as the broadest carbon-utilization diversity, while strain R1Gly possesses the most siderophore-related genes and demonstrated endophytic switchgrass growth promotion despite its origin of isolation (the tobacco endosphere). While there were differences among all strains, strains F10Cl (isolated from switchgrass) and R1Gly (isolated from tobacco) demonstrated the highest functional similarity, suggesting that, while endophyte community structure may vary widely based on host species, differences in functional diversity are not as clearly delineated.

Overall, the results of this study not only support previous research demonstrating the diversity of endophytic plant growth promotion relationships with hosts, but also indicate that there is diversity in PGP characteristics colonizing a single cultivar. Future studies should compare the effects of diverse endophytes on PGP and plant-bacteria interactions *in planta* to further investigate the different effects of endophytes with diverse PGP traits on plant resilience. Furthermore, we demonstrated colonization and growth promotion of a monocot host by a dicot-derived endophyte, suggesting the ability for directed infection of endophyte consortia in a broad range of hosts. Future studies should continue to evaluate the effects of both single, non-model endophytes and endophytic communities on plant robustness to determine if cultivation of specific endophytic communities with certain interactions can be utilized to improve the growth and resilience of various hosts under N limitation.

## Data Availability Statement

The datasets presented in this study can be found in online repositories. The names of the repository/repositories and accession number(s) can be found in the article/Supplementary Material.

## Author Contributions

RC obtained funding and designed the project and experiment. MS, SG-D, and RW conducted experiments and analyzed results. RC, SG-D, MS, and RW edited manuscript. All authors contributed to the article and approved the submitted version.

## Conflict of Interest

The authors declare that the research was conducted in the absence of any commercial or financial relationships that could be construed as a potential conflict of interest.

## Publisher’s Note

All claims expressed in this article are solely those of the authors and do not necessarily represent those of their affiliated organizations, or those of the publisher, the editors and the reviewers. Any product that may be evaluated in this article, or claim that may be made by its manufacturer, is not guaranteed or endorsed by the publisher.

## Footnotes

1
http://www.ncbi.nlm.nih.gov/Genbank
