# Supplementary material for: Plant Growth Promotion Diversity in Switchgrass-Colonizing, Diazotrophic Endophytes
Source: Front Microbiol. 2021 Nov 12;12:730440. doi: 10.3389/fmicb.2021.730440 (PMC8633415; doi:10.3389/fmicb.2021.730440)
Supplement: Supplementary file 1 [file Data_Sheet_1.PDF]

Isolate	Closest species match	NCBI Identity	Silva Identity	Accession	Ref.
F10Cl	*Klebsiella variicola* strain GJ3	99.9%	99.9%	SAMN 05361855	(Di et al., 2017)
R1C	*Azospirillum agricola* strain CC-HIH038	98.0%	97.9%	NR 148768	(Lin et al., 2016)
R1Gly	*Raoultella terrigena* strain ATCC 33257	99.6%	99.4%	NR 114503	(Drancourt et al., 2001)
