# Supplementary material for: Plant Growth Promotion Diversity in Switchgrass-Colonizing, Diazotrophic Endophytes
Source: Front Microbiol. 2021 Nov 12;12:730440. doi: 10.3389/fmicb.2021.730440 (PMC8633415; doi:10.3389/fmicb.2021.730440)
Supplement: Supplementary file 2 [file Data_Sheet_2.PDF]

Plant growth promotion diversity in switchgrass-colonizing, diazotrophic endophytes

Supplemental Information

Authors: Sara Gushgari-Doyle[1], Marcus Schicklberger[1], Yifan Li[1], Robert Walker[1], Romy Chakraborty[1]*

Affiliation: [1]Climate and Ecosystem Sciences, Earth and Environmental Sciences Area, Lawrence Berkeley National Laboratory, Berkeley, CA, USA

Table S1. Media recipe for modified Jensen's media

Modified Jensen's media recipe

Component	Amount/ 1L medium
Milli-Q water	980 mL
Sucrose	20 g
K_2HPO_4	1.0 g
KH_2PO_4	0.05 g
$MgSO_4$	0.5 g
NaCl	0.5 g
Fe_2SO_4	0.1 g
$NaMoO_4$ x H_2O	0.005 g
$CaCO_3$	2.0 g
Mineral mix	10 mL
Vitamin mix	10 mL

Mineral mix

Component	100x Stock concentration (mg/L)	Final concentration (mg/L)
Na_2EDTA	2501.184	25.012
$MgSO_4$ x $7H_2O$	24893.470	248.935
$MnSO_4$	19.026	0.190
NaCl	58.440	0.584
$FeCl_2$ x $2H_2O$	107.357	1.074
$CaCl_2$ x $2H_2O$	7130.50	71.305
$CoCl_2$	118.965	1.190
$ZnSO_4$	29.904	0.299
$CuSO_4$ x $5H_2O$	5.000	0.050
H_3BO_3	350.920	3.509
Na_2MoO_4 x $2H_2O$	93.635	0.936
$NiCl_2$ x $6H_2O$	118.845	1.188
Na_2SeO_4	28.350	0.284

Vitamin mix

Component	100x Stock concentration (mg/L)	Final concentration (mg/L)
d-Biotin	200.0	2.0
Folic Acid	200.0	2.0
Pyridoxine HCl	1000.0	10.0
Riboflavin	500.0	5.0
Thiamine	500.0	5.0
Nicotinic Acid	500.0	5.0
Pantothenic Acid	500.0	5.0
Vitamin B12	10.0	0.1
p-Amino Benzoic Acid	500.0	5.0
D,L-6,8-Thiotic Acid	500.0	5.0

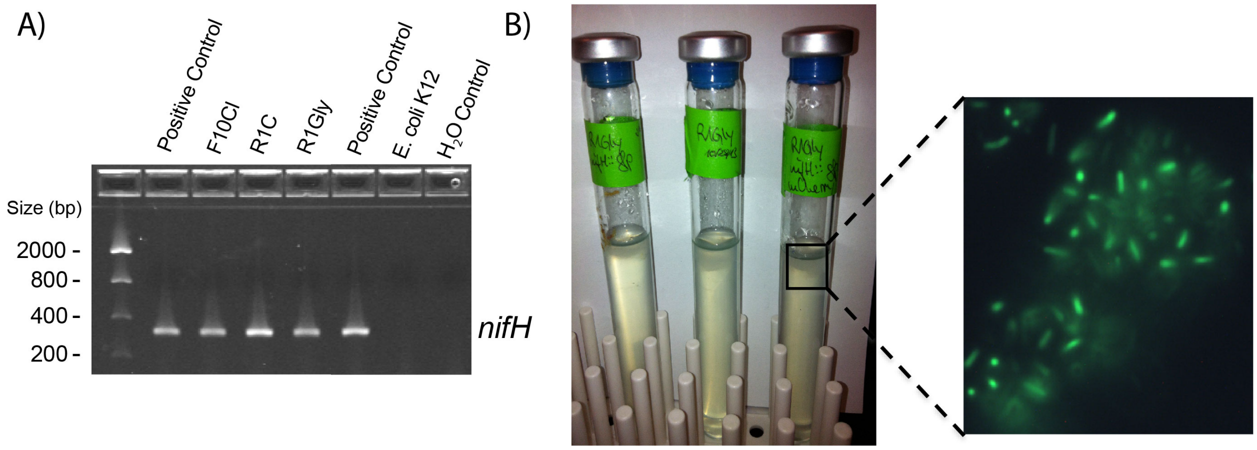

Figure S1. (A) Product of 300bp fragment of *nifH* gene amplified with PolFR primers in each isolate (B) Fluorescent image of R1Gly *nifH::gfp* grown on semi-solid medium under oxygen-limiting conditions, indicating active N-fixation.

Table S2. Primers used for synthesis of strain R1Gly-nifH::gfp.

Primer name	Sequence	Function
bac_nifH_for	5' - GGCTGCGATCCVAAGGCCGAYTCVACCCG	primer walking, confirmation of insertion
nifD_rev	5' - CCTTGATGCTGTCGTCGAACAGAGC	primer walking, confirmation of insertion
gfp_for	5' - GGGGAAGGAGATATACATATG	amplification of *gfp* from pMQ97
gfp_rev	5' - ATGCCTGGCAGTTTATGG	amplification of *gfp* from pMQ97
pMQ_nifHR1Gly_up_for	5' - TGACCATGATTACGAATTCGAGCTCGGTACCCGGGCGGGGACGTGGTCTGCGG	amplification of upstream fragment, fusion of gfp with upstream fragment
gfp_nifHR1Gly_up_rev	5' - CATATGTATATCTCCTTCCCCTCAGGCCGCGTTTTCTTC	amplification of upstream fragment
gfp_nifHR1Gly_down_for	5' - CCGCCATAAACTGCCAGGCATGCACAGGACAATCATCATG	amplification of downstream fragment
pMQ_nifHR1Gly_down_rev	5' - ACGGCCAGTGCCAAGCTTGCATGCCTGCAGGTCGACATCGCCGATAAGCCCCAC	amplification of downstream fragment, fusion of gfp with downstream fragment
pMQ_test_for	5' - CTTTACACTTTATGCTTCCG	confirmation of fragment of interest in pMQnifH::gfp
pMQ_test_rev	5' - CGATTAAGTTGGGTAACG	confirmation of fragment of interest in pMQnifH::gfp

Table S3. Genome characteristics of isolated switchgrass diazotrophic endophytes.

Attribute	Strain F10Cl		Strain R1C		Strain R1Gly	
	Value	% of Total	Value	% of Total	Value	% of Total
Genome size (bp)	5,734,768	100	7,770,695	100	5,717,197	100
DNA coding (bp)	5,106,120	89.04	6,947,217	89.4	5,089,652	89.02
DNA G+C (bp)	3,269,780	57.02 [1]	5,395,111	69.43 [1]	3,306,646	57.84 [1]
DNA scaffolds	3	100	8	100	10	100
Total genes	5,537	100	6,893	100	5,456	100
Protein coding genes	5,327	96.21	6,780	98.36	5,239	96.02
RNA genes	210	3.79	113	1.64	217	3.98
Pseudo genes	77	1.39 [2]	102	1.48 [2]	77	1.41 [2]
Genes in internal clusters	743	13.24	1,406	20.4	700	12.83
Genes with function prediction	4,617	83.38	5,450	79.07	4,635	84.95
Genes assigned to COGs	4,273	77.17	4,747	68.87	4,320	79.18
Genes with Pfam domains	4,879	88.12	5,626	81.62	4,884	89.52
Genes with signal peptides	537	9.7	578	8.39	532	9.75
Genes with transmembrane helices	1,328	23.98	1,534	22.25	1,315	24.1
CRISPR repeats	1		7		0	

[1] GC percentage shown as count of G's and C's divided by the total number of bases. The total number of bases is not necessarily synonymous with a total number of G's, C's, A's, and T's.

[2] Pseudogenes may also be counted as protein coding or RNA genes, so are not additive under total gene count.

Table S4. Summary of plasmids and putative plasmids found in the genomes of stains F10Cl, R1C, and R1Gly.

R1C							
GC content	69.4						
Scaffold	GC content	length (kbp)	no. genes	PLSDB result	match accession	distance	p-value
A. lipoferum R1C : Ga0048941_13	65.06	214.5	165		NZ_CP039651.1	0.1823	4.79E-58
A. lipoferum R1C : Ga0048941_16	68.36	263.8	229		NC_013855.1	0.1917	5.97E-45

F10Cl							
GC content	57.0						
Scaffold	GC content	length (kbp)	no. genes	PLSDB result	match accession	distance	p-value
K. variicola F10Cl : JN20DRAFT_unitig_3_quiver.1	46.01	280.9	291	pNDM-MAR	JN420336.1	0.0346	0

R1Gly							
GC content	57.9						
Scaffold	GC content	length (kbp)	no. genes	PLSDB result	match accession	distance	p-value
R. terrigena R1Gly : IE84DRAFT_unitig_0_quiver.7	54.84	42.5	43		NC_021742.1	0.1002	0
R. terrigena R1Gly : IE84DRAFT_unitig_18_quiver.4	51.92	35.9	38		NC_021742.1	0.1744	4.64E-80
R. terrigena R1Gly : IE84DRAFT_unitig_2_quiver.1	48.26	71.7	82	IncFII	NZ_CP041050.1	0.2109	6.51E-34

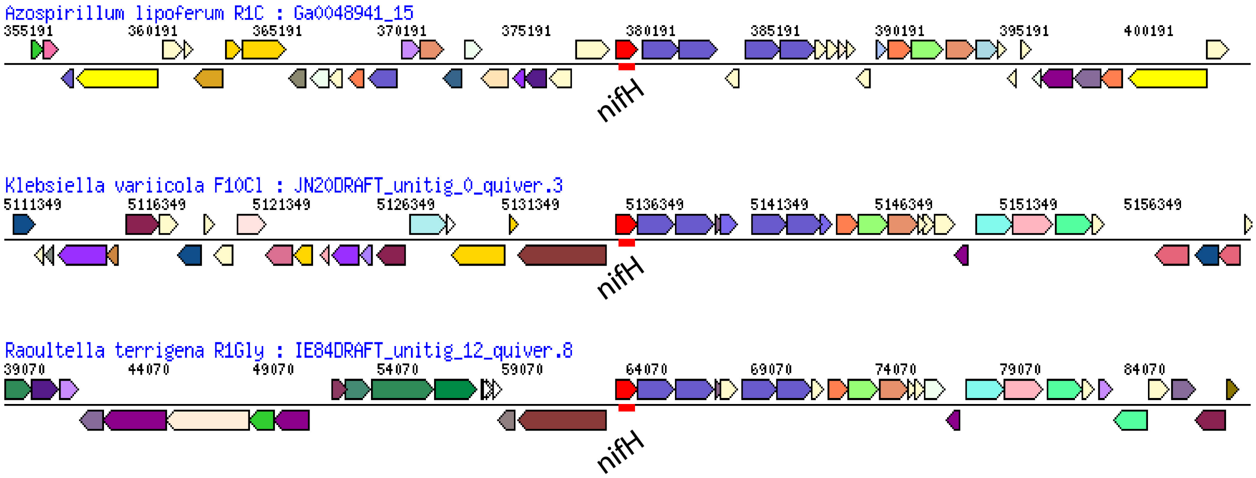

Figure S2. Genetic context of the nif operon, highlighting nifH (red) in each isolate. Other protein-coding genes are colored by COG.

Table S5. Genome loci of genes encoding plant growth-promoting traits in strains R1C, F10Cl, and R1Gly.

	R1C	F10Cl	R1Gly
**N-fixation**			
N-fixation protein NifB	Ga0048941_4585	JN20DRAFT_5505	IE84DRAFT_4254
N-fixation protein NifD	Ga0048941_3311	JN20DRAFT_5489	IE84DRAFT_4238
N-fixation protein NifE	Ga0048941_3314	JN20DRAFT_5493	IE84DRAFT_4242
N-fixation protein NifH	Ga0048941_3310	JN20DRAFT_5488	IE84DRAFT_4237
N-fixation protein NifK	Ga0048941_3312	JN20DRAFT_5490	IE84DRAFT_4239
N-fixation protein NifL		JN20DRAFT_5503	IE84DRAFT_4252
N-fixation protein NifN	Ga0048941_3315	JN20DRAFT_5494	IE84DRAFT_4243
N-fixation protein NifT	Ga0048941_4578	JN20DRAFT_5491	IE84DRAFT_4240
N-fixation prote NifQ	Ga0048941_4645	JN20DRAFT_5506	IE84DRAFT_4255
N-fixation protein NifU	Ga0048941_1988		
N-fixation protein NifX	Ga0048941_3316	JN20DRAFT_5492	IE84DRAFT_4244
		JN20DRAFT_5495	
N-fixation protein NifZ	Ga0048941_4580	JN20DRAFT_5500	IE84DRAFT_4249
	Ga0048941_4581		
N-fixation protein AnfH			IE84DRAFT_2787
N-fixation protein AnfO			IE84DRAFT_2793
N-fixation protein FixG	Ga0048941_0960		
	Ga0048941_1588		
	Ga0048941_2682		
	Ga0048941_4175		
N-fixation protein FixH	Ga0048941_4176		
N-fixation protein FixI	Ga0048941_4177*		
**Fe mobilization**			
Siderophore transport protein TonB	Ga0048941_1017	JN20DRAFT_4989	IE84DRAFT_0039
		JN20DRAFT_5110	IE84DRAFT_3777
			IE84DRAFT_4512
			IE84DRAFT_4694
			IE84DRAFT_4984
Siderophore transport protein ExbB	Ga0048941_1019	JN20DRAFT_1481	IE84DRAFT_3536
			IE84DRAFT_3775
Siderophore transport protein ExbD	Ga0048941_1018	JN20DRAFT_1480	IE84DRAFT_3537
			IE84DRAFT_3778
Catecholate TonB-dependent receptor	Ga0048941_1016	JN20DRAFT_4137	IE84DRAFT_1775
Dicitrate TonB-dependent receptor			IE84DRAFT_2204
Enterobactin exporter EntS family	Ga0048941_2556	JN20DRAFT_3422	IE84DRAFT_1574
Enterobactin receptor		JN20DRAFT_2332	
		JN20DRAFT_3415	
		JN20DRAFT_4131	
Siderophore biosynthesis EntD			IE84DRAFT_5008
Siderophore reductase	Ga0048941_6714		IE84DRAFT_3485
	Ga0048941_6755		
Enterobactin synthase		JN20DRAFT_3414	IE84DRAFT_1566
		JN20DRAFT_3418	IE84DRAFT_1570
Pyochelin synthase			IE84DRAFT_5006
Mycobactin calicyl-AMP ligase			IE84DRAFT_5001

**P mobilization**			
Acid phosphatase	Ga0048941_2777	JN20DRAFT_2326	IE84DRAFT_0306
		JN20DRAFT_4093	IE84DRAFT_2343
4-phytase		JN20DRAFT_3441	IE84DRAFT_4329
**IAA biosynthesis**			
Indolepyruvate decarboxylase		JN20DRAFT_0798	IE84DRAFT_3106
Aldehyde dehydrogenase (NAD+)	Ga0048941_0254		IE84DRAFT_1958
	Ga0048941_0426		IE84DRAFT_3043
	Ga0048941_1157		
	Ga0048941_1168		
	Ga0048941_1929		
	Ga0048941_2084		
	Ga0048941_6550		
	Ga0048941_6634		
Amidase	Ga0048941_2821	JN20DRAFT_4569	IE84DRAFT_1541
	Ga0048941_5936		IE84DRAFT_3825
Aminotransferase		JN20DRAFT_4253	
Nitrile hydratase		JN20DRAFT_4567	
		JN20DRAFT_4568	
**Putresciene biosynthesis**			
Ornithine decarboxylase	Ga0048941_3608	JN20DRAFT_1464	IE84DRAFT_3642
	Ga0048941_5335		
	Ga0048941_6042		
**Spermine/sperimidine biosynthesis**			
Spermidine synthase		JN20DRAFT_2886	IE84DRAFT_5198
Spermine/spermidine n-acetyltransferase		JN20DRAFT_4426	IE84DRAFT_4391
**Beta-glucanase**			
Beta-glucanase	Ga0048941_2167		
	Ga0048941_2586		
	Ga0048941_3983		
	Ga0048941_4770		
**Chitinase**			
Chitinase		JN20DRAFT_4087	IE84DRAFT_2338
**Cellulase**			
Cellulase			IE84DRAFT_0302
**Alpha-amylase**			
Alpha-amylase	Ga0048941_4711	JN20DRAFT_2059	IE84DRAFT_0854
	Ga0048941_4976	JN20DRAFT_4571	IE84DRAFT_4945
		JN20DRAFT_4736	
		JN20DRAFT_5369	

Table S6. Organic carbon compounds supporting growth of R1C, F10Cl, and R1Gly. A (+) indicates growth was observed while a blank cell indicates no growth was observed.

	R1C	F10Cl	R1Gly
Sucrose	+	+	+
Fructose	+	+	+
Glucose			+
Ribose	+	+	
Xylose	+	+	
Lyxose	+	+	
Cellobiose		+	+
Arabinose		+	+
Mannose	+	+	+
Rhamnose	+	+	+
Maltose		+	+
Malic Acid		+	+
Citric Acid	+		+
Lactic Acid		+	+
Benzoic Acid			
Acetic Acid		+	+
Oxalic Acid	+		+
Succinic Acid		+	+
Fumaric Acid		+	+
Galacturonic Acid		+	+
Glycolic Acid		+	
Mucic Acid		+	+
Phytic Acid		+	+
Nicotinic Acid		+	
Urea		+	

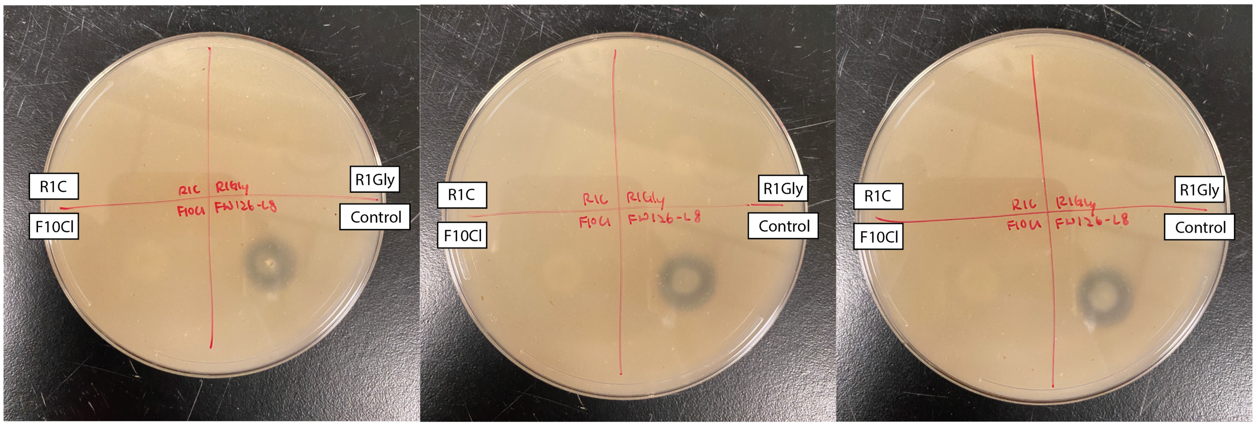

Figure S3. Chitinase activity agar plate assay exhibiting the absence of chitinolytic activity in strains R1C, F10Cl, and R1Gly as compared to *Cohnella phaseoli* strain FW126-L8, the positive

control (in experimental triplicate). The dark blue discoloration observed in the positive control indicates chitinolytic activity.

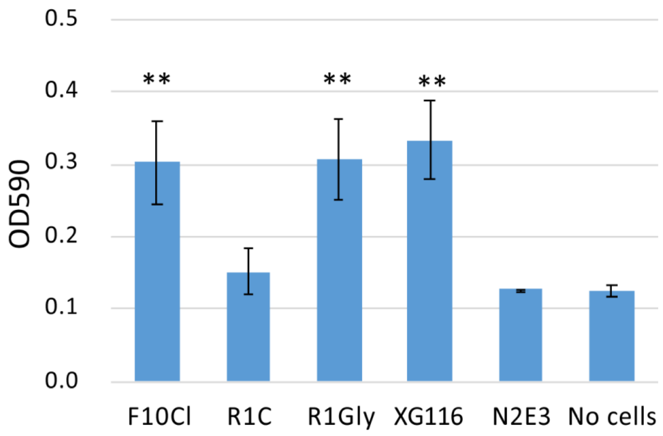

	Average	Standard Dev	Alpha
F10Cl	0.30	0.056	0.0028
R1C	0.15	0.033	0.12
R1Gly	0.31	0.056	0.0025
XG116	0.33	0.054	0.0014
N2E3	0.13	0.0010	0.35
No cells	0.13	0.0068	0.50

Figure S4. Results from cellulase activity assay. Error bars and "Standard Dev" indicate one standard deviation of three experimental triplicates and "Alpha" indicates the result of a one-sided, unpaired Student-T test. Asterisks denote statistical significance. *Cellulomonas pakistanensis* XG116 is included as positive control. Uninoculated medium (no cells) and *Pseudomonas fluorescens* strain N2E2 are included as negative controls.
